# Reverse vaccinology approach to identify novel and immunogenic targets against *Porphyromonas gingivalis*: An *in silico* study

**DOI:** 10.1371/journal.pone.0273770

**Published:** 2022-08-30

**Authors:** Omid Nasiri, Mahsa Hajihassani, Narjes Noori Goodarzi, Sepideh Fereshteh, Negin Bolourchi, Farzaneh Firoozeh, Omid Azizi, Farzad Badmasti

**Affiliations:** 1 Department of Bacteriology, Pasteur Institute of Iran, Tehran, Tehran, Iran; 2 Department of Pathobiology, School of Public Health, Tehran University of Medical Sciences, Tehran, Tehran, Iran; 3 Department of Microbiology, School of Medicine, Alborz University of Medical Science, Karaj, Alborz, Iran; 4 Health Sciences Research Center, Torbat-e Heydarieh University of Medical Sciences, Torbat-e Heydarieh, Razavi Khorasan, Iran; 5 Microbiology Research Center (MRC), Pasteur Institute of Iran, Tehran, Iran; University of Nebraska-Lincoln, UNITED STATES

## Abstract

*Porphyromonas gingivalis* is a primary causative agent of chronic periodontitis. Moreover, it leads to several systemic diseases, including rheumatoid arthritis, cardiovascular, neurodegenerative, and Alzheimer’s diseases. It seems that the development of a vaccine against this bacterium is necessary. Thus, this study decided to identify novel immunogenic targets and developed multiple epitope-based vaccines against *P*. *gingivalis*. For this purpose, the pan/core-proteome of this bacterium was studied, and the suitable vaccine targets were selected based on different properties, including exposed localization of proteins, antigenicity, non-allergenicity, non-similarity to host proteome, stability, B-cell epitopes and MHC II binding sites, sequence conservation, molecular docking, and immune simulation. Through the quartile scoring method, 12 proteins with ≥ 20 scores were considered as suitable immunogenic targets. The results of the protein domain and functional class search showed that most of the immunogenic proteins were involved in the transport and metabolism of inorganic ions and lipids. In addition, two unknown function proteins, including WP_004584259.1 and WP_099780539.1 were detected as immunogenic targets. Three constructions carrying multi-epitopes were generated including Naked, LCL, and as chimeric structures. Among them, FliC chimeric protein had the strongest affinity to the human TLR2, 4, and 6, while the LCL platform represented the highest level of immune stimulation response. The obtained results from this study revealed new insights into prophylactic routes against *P*. *gingivalis* by introducing novel immunogenic targets. However, further investigations, including site-directed mutation and immunoassay are needed to confirm the pathogenic role and protectivity of these novel proteins.

## 1. Introduction

Periodontitis is an inflammatory polymicrobial disease and one of the humans’ most common bacterial infections affecting nearly 50% of the global population [1]. The pathological process in periodontitis includes persistent bacterial colonization coupled with a self-damaging host immune response that leads to hard/soft tissue destruction of structures supporting the tooth and is the leading cause of tooth loss in adults [2]. The development of periodontitis is a multifactorial process involving interactions between the host and the microorganisms, especially *Porphyromonas gingivalis*, *Treponema denticola*, and *Tannerella forsythia* that colonize the oral cavity [3]. *P*. *gingivalis* is a nonmotile, Gram-negative, obligately anaerobic, and rod-shaped bacterium that colonizes dental plaque biofilms in the human oral cavity, and is considered the primary causative agent responsible for the development of chronic periodontitis [1]. This organism is the focus of research studies due to its capacity to evade immune system responses and is the most active periodontal pathogen [4]. It is closely associated with the occurrence and development of numerous diseases, including atherosclerosis, cancer, and Alzheimer’s disease.

*P*. *gingivalis* has several virulence factors, including cysteine proteases (gingipains), heat shock proteins, lipopolysaccharide, major fimbriae, and capsule. The gingipains can degrade several host proteins, such as complement proteins, immunoglobulins, cytokines/chemokines, and host cell receptors [5].

Several classes of antibiotics have been proposed to treat infections associated with *P*. *gingivalis*. However, in recent years, concerns have been raised about the efficacy of antimicrobials in treating infections associated with oral biofilm. Several studies have demonstrated *P*. *gingivalis* can survive antibiotic treatment and leads to the recurrence of chronic periodontitis [6, 7]. The potential ability of bacteria to invade host cells is considered to be a mechanism that helps bacteria survive during antibiotics treatment. For these reasons, the development of an effective vaccine against periodontitis is highly desirable [4]. Vaccination can be a powerful strategy to combat severe infections as well as antimicrobial resistance.

With the advent of genome sequencing technology, a considerable revolution in immunization has occurred. Genomic databases have greatly facilitated the investigation of immunogenic candidates to develop new potential vaccine targets against pathogenic microorganisms [8]. Reverse vaccinology is a novel computational approach that exploits all the available data about the pathogen sequentially to identify the most suitable targets for vaccine design and development. This approach reduces the period of vaccine candidate detection and evaluation [9]. The strategy aims to combine bioinformatics with immunogenetics and immunogenomics for the development of novel vaccine targets [10]. Computationally, designed vaccines are proven their effectiveness, safety, specificity, and thermodynamically stability compared to conventional approaches to vaccine development [11]. This research was performed to design prophylactic vaccine targets in *P*. *gingivalis* by employing *in silico* approaches.

## 2. Materials and methods

### 2.1. Retrieval of primary data and pan/core-genome analysis

In this study, 17 *P*. *gingivalis* strains with completely annotated genome sequences were retrieved from the GenBank database (https://www.ncbi.nlm.nih.gov/genbank/) and translated by CLC Genomics Workbench software (Qiagen, Hilden, Germany). Pan/core-genome analysis was performed by the Bacterial Pan Genome Analysis tool (BPGA) [12] the core-proteome was determined with a cut-off > 0.5. The core, accessory, and unique proteins distribution among metabolic pathways were compared using the Kyoto Encyclopedia of Genes and Genomes (KEGG) database.

### 2.2. Subcellular localization of putative proteins

The subcellular localization of the proteins was identified using PSORTb version 3.0.3 (https://www.psort.org/psortb/). Only outer membranes, extracellular, and secreted proteins were selected in this step [13].

### 2.3. Determination of antigenicity and allergenicity of putative immunogenic targets

The antigenicity of the putative immunogenic targets was predicted using the VaxiJen tool (http://www.ddg-pharmfac.net/vaxijen/VaxiJen/VaxiJen.html) with a cut-off value ≥ 0.5 [14]. Subsequently, the allergenicity of the antigenic proteins was determined using the AlgPred 2.0 web tool (http://crdd.osdd.net/raghava/algpred/) with a cut-off ≥ 0.5 [15].

### 2.4. Sequence similarity of proteins with the human proteome

All selected proteins were analyzed to determine sequence similarity to the human proteome (Humo sapiens, taxid: 9606) using the PSI-BLAST tool in the BLASTp database (https://blast.ncbi.nlm.nih.gov/Blast.cgi?PAGE=Protein) [16]. Sequence similarity detection with PSI-BLAST is more sensitive than the regular BLAST if they are distantly related to the query sequence. Proteins that showed similarity with coverage of ≥ 30% and identity of ≥ 25% were excluded from the analysis.

### 2.5. Detection of linear B-cell epitopes and human MHC-II binding sites

Linear B-cell epitopes of all selected putative immunogenic proteins from the previous steps were identified with a threshold ≥ 0.6 using the BepiPred v2.0 tool (https://services.healthtech.dtu.dk/service.php?BepiPred-2.0) [17]. The ratio of B-cell epitopes to the total number of amino acids was calculated for each protein. On the other hand, human MHC-II binding sites were predicted using the IEDB resource TepiTool (http://tools.iedb.org/tepitool/) with a cut-off of the top 5% of peptides [18]. The ratio of MHC-II binding sites to the total number of amino acids was calculated for all proteins.

### 2.6. Physicochemical properties of putative immunogenic proteins

Physicochemical properties of proteins were analyzed using different databases. The functional class of the proteins was determined by the VICMpred database (https://webs.iiitd.edu.in/raghava/vicmpred/submission.html). The number of amino acids, molecular weight, theoretical pI value, estimated half-life, aliphatic index, and instability index were determined using the Expasy ProtParam server (https://web.expasy.org/protparam/) [19]. The instability index provides an estimate of the stability of the protein in a test tube. There are particular dipeptides with different occurrences in the stable and unstable proteins. To compute the instability index, a weight value of instability is assigned to each of the 400 different dipeptides [20]. In addition, the adhesion probability was determined using the Vaxign database (http://www.Violinet.org/vaxign2). Adhesins are potential vaccine candidates due to their role in adherence, colonization, and bacterial survival [21].

### 2.7. Quartile scoring method

The selected proteins were analyzed using the quartile method scoring using eight indicators, including functional class (virulence, cellular process, metabolic molecule, and unknown), antigenicity, hydropathy index, instability index, MHC-II binding site ratio, linear B-cell epitope ratio, conformational B-cell epitope, and adhesion probability value. The sum of all scores for each protein was considered the final score. Proteins with ≥ 20 points were considered suitable immunogenic targets [22].

### 2.8. Prediction of tertiary structure and characterization of conformational B-cell epitopes

The tertiary structure (3D) of the putative immunogenic proteins was characterized using the Robetta tool (https://robetta.bakerlab.org/) [23]. The quality of the 3D model was checked using the ProSA-web server (https://prosa.services.came.sbg.ac.at/prosa.ph). This server displays the potential errors in the 3D model [24]. In addition, ElliPro (http://tools.iedb.org/ellipro/) was used to identify the conformational B-cell epitopes with a threshold value of ≥ 0.8. The predicted conformational B-cell epitopes were displayed on the surface of each protein in different colors using Jmol software (It should be noted that only surface-exposed epitopes, detected by PRED-TMBB, were displayed).

### 2.9. Protein domain search

Conserved Domain Database, CDD (https://www.ncbi.nlm.nih.gov/Structure/cdd/cdd.shtml), and EggNOG (http://eggnog5.embl.de/#/app/home) were used to find the major protein domains. CDD is part of NCBI’s Entrez query and provides an annotation of protein sequences with the location of the conserved domain [25]. EggNOG is a hierarchical, functionally and phylogenetically annotated orthology resource based on 5090 organisms and 2502 viruses [26].

### 2.10. Protein-protein interaction networks

In this part, we used the STRING database (https://string-db.org/) to understand the interactions of putative vaccine candidates with unknown functions with other proteins of *P*. *gingivalis* to estimate their function. The connection scores > 0.5 were considered.

### 2.11. Determining conservation of B-cell epitopes

Linear B-cell epitopes were predicted using the BepiPred database (Threshold ≥ 0.6). The conformational epitopes are obtained using the ElliPro database (Threshold ≥ 0.8). The Conservancy of linear and conformational B-cell epitopes was determined among 17 *P*. *gingivalis* strains with complete annotation at GenBank (https://www.ncbi.nlm.nih.gov/genbank) [27]. Finally, epitopes with conservation less than 80% and antigenicity less than 1 were excluded from this study.

### 2.12. Construction of multiple epitope-based vaccines

Three multiple epitope-based vaccines were generated using three different platforms including Naked, FliC, and LCL. Different arrangements of the selected epitopes with rigid (EAAAK) and flexible (GPGPG) linkers were developed, and the most antigenic models were chosen. The tertiary structures (3D) of the Naked, FliC, and LCL chimeras were modeled using the Robetta web-tool. The 3D structures were validated by the ProSA-web server and Ramachandran diagrams.

### 2.13. Molecular docking and immune simulation

Molecular docking and the binding affinity of multi-epitope-based vaccines to human TLR-1 (PDB: 2Z7X), TLR-2 (PDB: 2Z7X), TLR-4 (PDB: 3FXI), and TLR-6 (PDB: 379A) was investigated using pyDockWEB (https://life.bsc.es/pid/pydockweb/default/index) [28]. In addition, C-ImmSim (https://kraken.iac.rm.cnr.it/C-IMMSIM/index.php) was used to predict immune simulations of multi-epitope-based vaccines.

## 3. Results

### 3.1. Pan/core-genome analysis

**Fig 1:**
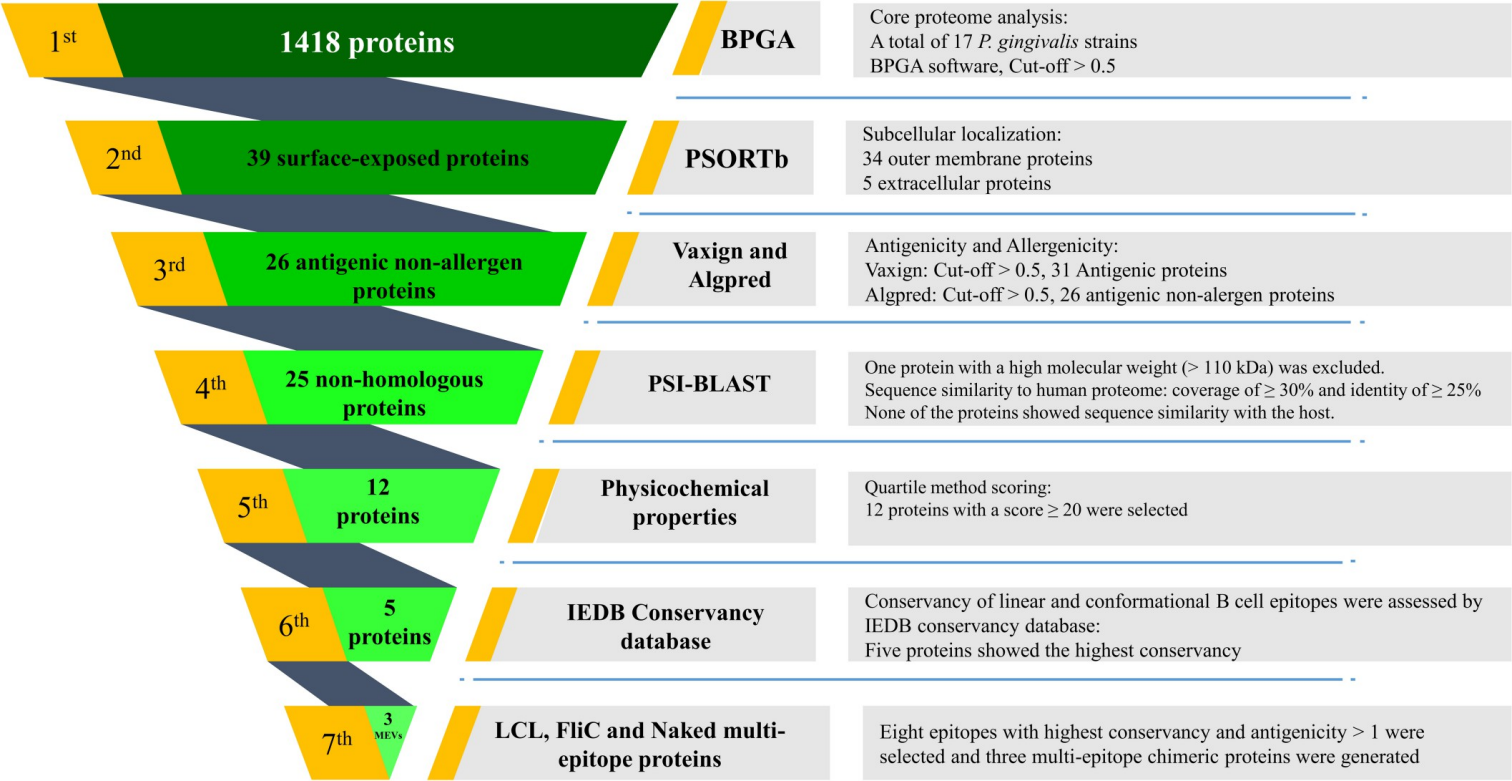
Schematic representation of the selection and validation of novel putative immunogenic targets against *P*. *gingivalis* using a reverse vaccinology approach. All criteria and thresholds are shown in the flowchart. MEVs: Multi-epitope vaccines.

The workflow to identify novel immunogenic targets against *P*. *gingivalis* is presented in Fig 1.

**Fig 2:**
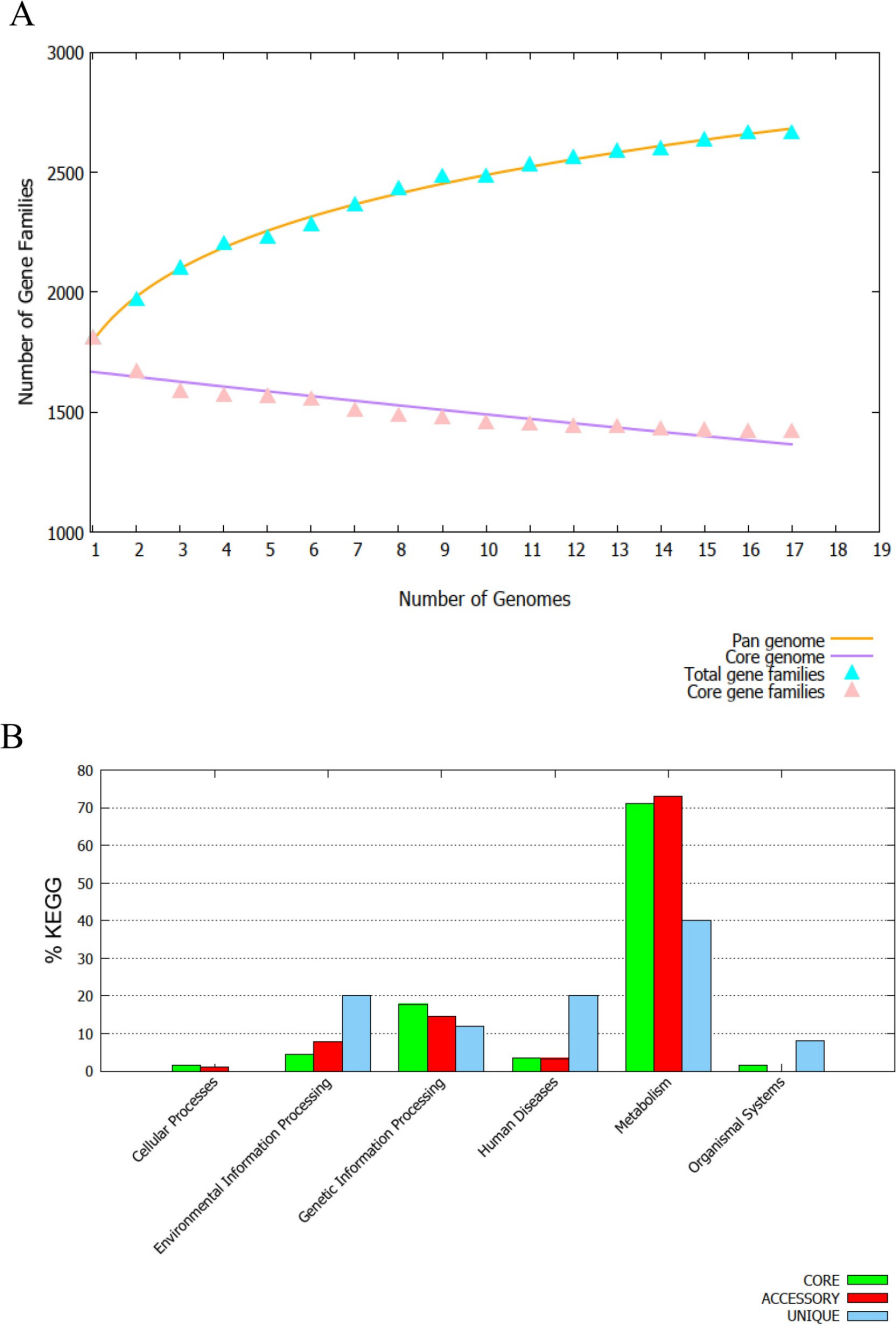
**A) Demonstration of the core-pan plot between 17 *P*. *gingivalis* strains with a cut-off > 0.5.** The pan and core-proteome consist of 1985 and 1418 proteins, respectively. The X-axis of this figure shows the number of strains, and the Y-axis of Fig 2A shows the percentage of core- and pan- genes among strains. Blue triangles: number of total gene families. Pink triangles: number of core gene families. **B) The distribution of the core, accessory and unique proteins among metabolic pathways was compared using the Kyoto Encyclopedia of Genes and Genomes (KEGG) database**. The X-axis shows different categories of proteins, and the Y-axis shows the percentage (proportion) of proteins in each category. The majority of core proteins were involved in metabolism, followed by genetic information processing, and environmental information processing.

The core-pan plot showed that the pan-proteome and core-proteome of *P*. *gingivalis* consist of 1985 and 1418 proteins, respectively. Based on the KEGG mapping of core, accessory, and unique genes among different *P*. *gingivalis* strains, six different categories were introduced. The majority of core proteins were involved in metabolism, followed by genetic information processing and environmental information processing. See Fig 2.

### 3.2. Identification of antigenic, non-allergen, non-homologous to human and surface-exposed proteins

Surface-exposed and secreted proteins are more easily represented in the immune system and are capable of inducing a robust immune response. Thus, a total number of 39 surface-exposed proteins, including 35 Outer membrane Proteins (OMPs) and five extracellular proteins, were identified through subcellular localization analysis. Of the 39 proteins selected in the previous step, eight proteins were non-antigen. Moreover, among 31 antigenic proteins, five proteins were identified as allergens, so they were excluded from this study. Finally, 26 antigenic and non-allergenic proteins remained. PSI-BLAST analysis revealed no similarity between the human proteome and the 26 putative immunogenic proteins.

### 3.3. Characterization of immunogenic epitopes

**Fig 3:**
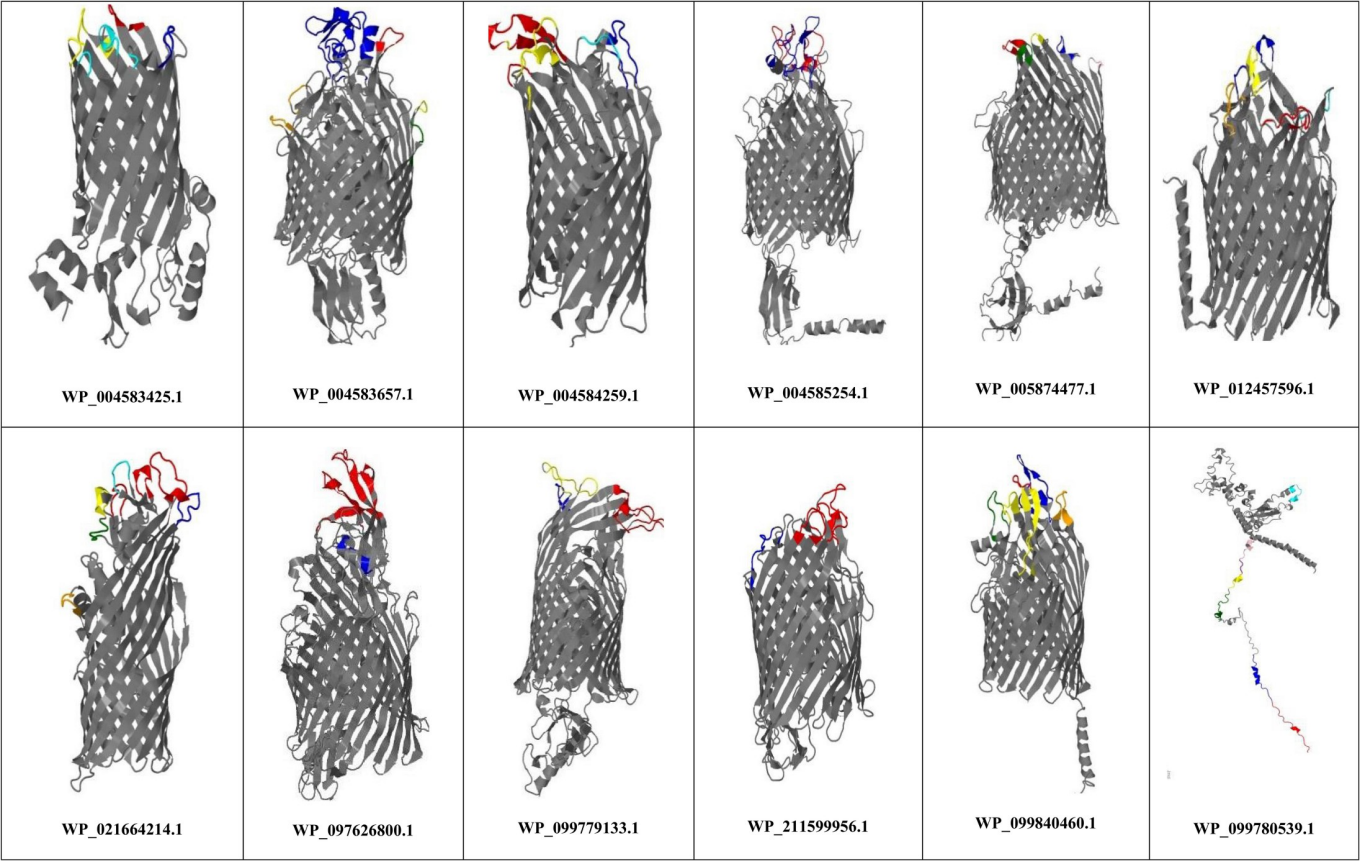
Surface-exposed conformational epitopes of prioritized proteins. The tertiary structures of the proteins were predicted by the Robetta web tool, and the surface-exposed epitopes were characterized on the 3D structure of proteins using Jmol software. The number of surface-exposed conformational epitopes of each protein is listed in parentheses: WP_099780539.1 (7 epitopes), WP_021664214.1 (6 epitopes), WP_099840460.1 (5 epitopes), WP_012457596.1 (5 epitopes), WP_005874477.1 (5 epitopes), WP_004583657.1 (5 epitopes), WP_004584259.1 (4 epitopes), WP_004583425.1 (4 epitopes), WP_099779133.1 (3 epitopes), WP_097626800.1 (2 epitopes), WP_004585254.1 (2 epitopes), and WP_211599956.1 (2 epitopes).

The number of linear and conformational B-cell epitopes, the ratio of B-cell epitopes, and the ratio of MHC-II binding sites of the 25 proteins were determined and presented in Supplementary Data 1. The sequences of linear and conformational B cell epitopes of each protein are presented in Supplementary Data 2. The number of linear and conformational B-cell epitopes were as follows: WP_097626800.1 (4 and 6 epitopes), WP_005874477.1 (4 and 10 epitopes), WP_004585254.1 (4 and 6 epitopes), WP_004583657.1 (4 and 8 epitopes), WP_099779133.1 (4 and 8 epitopes), WP_099840460.1 (4 and 8 epitopes), WP_211599956.1 (3 and 7 epitopes), WP_012457596.1 (4 and 10 epitopes), WP_021664214.1 (4 and 8 epitopes), WP_004584259.1 (2 and 8 epitopes), WP_099780539.1 (3 and 7 epitopes), and WP_004583425.1 (1 and 9 epitopes). The surface-exposed linear B-cell epitopes of the novel immunogenic targets are shown in Fig 3.

The linear and conformational B-cell epitopes were characterized, and the conservancy was determined and demonstrated in S2 Table. WP_005874477.1, WP_012457596.1, WP_021664214.1, and WP_004583425.1 had the most conserved linear and conformational B- cell epitopes. Finally, 12 outer membrane proteins, including: (WP_097626800.1, WP_005874477.1, WP_004585254.1, WP_004583657.1, WP_099779133.1, WP_099840460.1, WP_211599956.1, WP_012457596.1, WP_021664214.1, WP_004584259.1, WP_099780539.1, and WP_004583425.1) were characterized as putative immunogenic targets against *P*. *gingivalis*.

### 3.4. Finding the best immunogenic candidates based on quartile scoring method and physiochemical properties

The selected proteins were classified into three distinct functional classes. The majority of proteins were involved in the cellular process (12/25), followed by virulence factors (10/25), and metabolism molecules (3/25). The estimated half-life of all proteins was > 10h (*E*. *coli*, *in vivo*). In this step, nineteen proteins were stable, while six were not. To facilitate the expression and purification of vaccine candidates *in vitro*, only proteins with a molecular weight < 110 kDa were selected for further study. Out of 26 antigenic and non-allergenic proteins, one protein had a high molecular weight (> 110 kDa) and was excluded from the study. Additional physicochemical properties of the proteins are listed in S1 Table.

**Fig 4:**
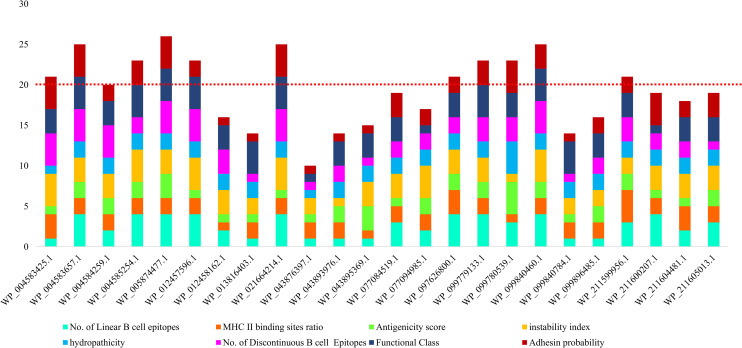
The comparative analysis of putative immunogenic targets against *P*. *gingivalis* based on quartile scoring method. Twelve proteins with a score ≥ 20 were selected: WP_005874477.1 (26), WP_004583657.1 (25), WP_021664214.1 (25), WP_099840460.1 (25), WP_004585254.1 (23), WP_012457596.1 (23), WP_099779133.1 (23), WP_099780539.1 (23), WP_004583425.1 (21), WP_097626800.1 (21), WP_211599956.1 (21), and WP_004584259.1 (20).

According to the quartile scoring method, 12 of 24 proteins with a score ≥ 20 were selected. The score of each protein was as follows: WP_005874477.1 (26), WP_004583657.1 (25), WP_021664214.1 (25), WP_099840460.1 (25), WP_004585254.1 (23), WP_012457596.1 (23), WP_099779133.1 (23), WP_099780539.1 (23), WP_004583425.1 (21), WP_097626800.1 (21), WP_211599956.1 (21), and WP_004584259.1 (20). See Fig 4. The physicochemical characteristics of 12 selected proteins are presented in Table 1.

**Table 1:** Physiochemical properties of 12 putative immunogenic proteins of *P*. *gingivalis*.

Accession number	Number of amino acids	Molecular weight (kDa)	Theoretical pI	Subcellular localization	Functional class	Adhesin probability	TMH	Estimated half time (*E*. *coli*)	Stability	Instability index	Aliphatic index	Hydropathicity	Allergenicity score	Similarity to human proteome	Antigenicity score	No. of linear B- cell epitopes	B-cell epitope ratio	Number of discontinuous B- cell epitope(s)	MHC-II binding sites ratio
WP_097626800.1	936	106.77	8.99	Outer membrane	Cellular process	0.325	1	>10 hours	stable	33.19	83.97	-0.369	0.12	No	0.601	10	0.162	5	0.208
WP_005874477.1	926	102.75	6.74	Outer membrane	Virulence factors	0.607	0	>10 hours	stable	34.85	72.55	-0.529	0.16	No	0.697	9	0.152	9	0.144
WP_004585254.1	848	94.28	5.53	Outer membrane	Virulence factors	0.482	0	>10 hours	stable	25	78.64	-0.393	-0.19	No	0.636	8	0.206	5	0.159
WP_004583657.1	833	92.73	5.74	Outer membrane	Virulence factors	0.645	0	>10 hours	stable	32.53	71.18	-0.465	-0.24	No	0.656	10	0.195	8	0.135
WP_099779133.1	827	92.42	9.2	Outer membrane	Virulence factors	0.551	1	>10 hours	stable	33.99	77.26	-0.328	-0.37	No	0.653	9	0.135	7	0.157
WP_099840460.1	745	83.48	6.42	Outer membrane	Virulence factors	0.553	0	>10 hours	stable	29.36	79.62	-0.328	0.25	No	0.629	9	0.178	8	0.163
WP_211599956.1	740	82.91	9.54	Outer membrane	Cellular process	0.403	1	>10 hours	unstable	44.95	77.93	-0.325	0.12	No	0.672	6	0.131	7	0.32
WP_012457596.1	646	73.12	8.9	Outer membrane	Virulence factors	0.378	0	>10 hours	stable	30.83	81.39	-0.359	0.12	No	0.531	8	0.108	10	0.171
WP_021664214.1	554	61	8.24	Outer membrane	Virulence factors	0.731	1	>10 hours	stable	30.1	69.42	-0.33	0.28	No	0.549	9	0.211	8	0.149
WP_004584259.1	547	62.60	9.17	Outer membrane	Cellular process	0.323	1	>10 hours	stable	35.27	79.4	-0.407	0.1	No	0.641	4	0.078	8	0.177
WP_099780539.1	401	46.11	9.45	Outer membrane	Cellular process	0.6	0	>10 hours	unstable	54.43	43.24	-1.154	0.24	No	0.868	7	0.403	7	0.069
WP_004583425.1	391	43.26	5.23	Outer membrane	Cellular process	0.59	0	>10 hours	stable	28.22	82.69	-0.142	0.33	No	0.539	3	0.117	8	0.23

### 3.5. Protein domain search and protein-protein interaction result

**Fig 5:**
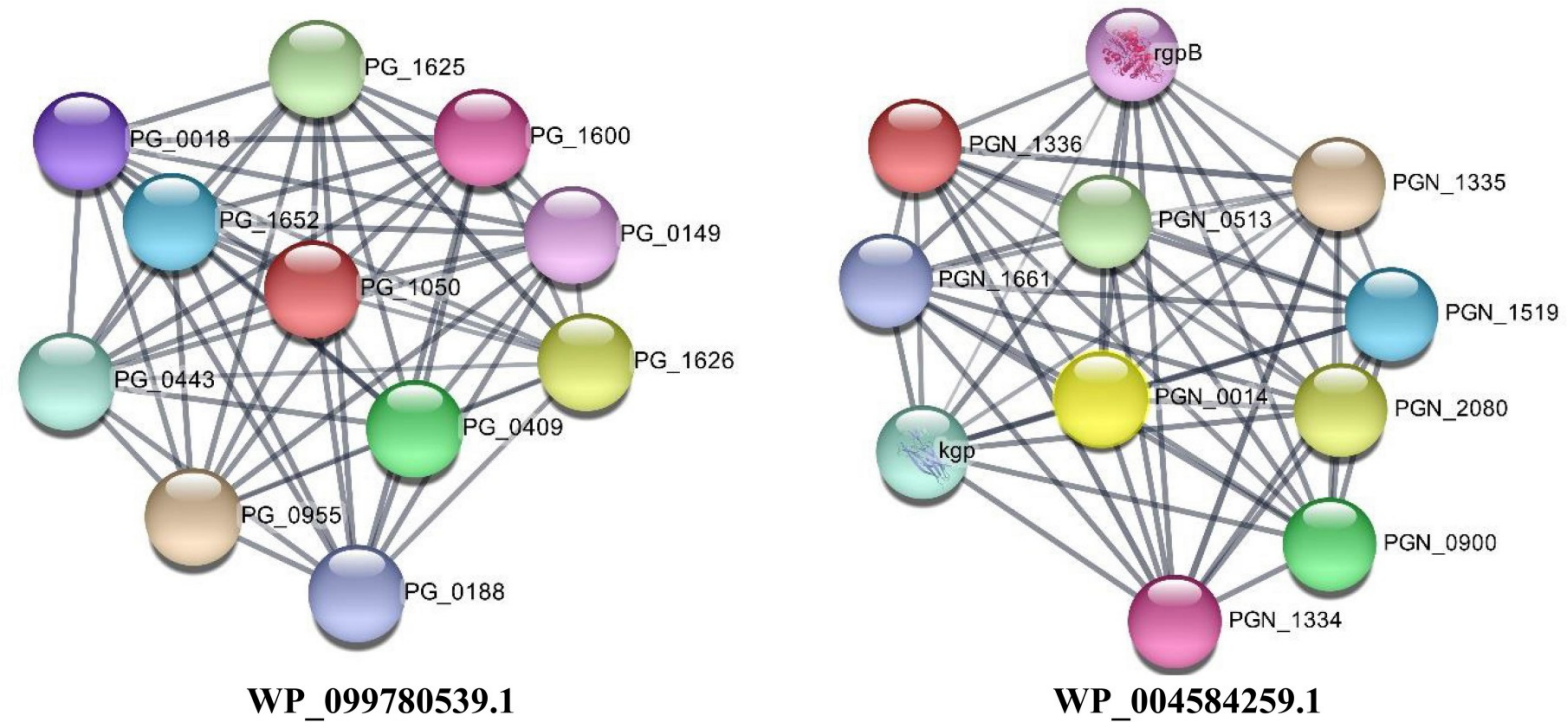
Protein-protein interaction networks of two hypothetical proteins (WP_099780539.1 and WP_004584259.1) with unknown functions with other proteins of *P*. *gingivalis*. WP_004584259.1 has neighborhood and co-occurrence interactions with TonB-dependent receptor (HR09_06515) and lipoproteins (HR09_06520).

The results of CDD and EggNOG showed that the studied proteins are involved in the transport and metabolism of inorganic ions and the transport and metabolism of lipids. However, the function of two proteins (WP_004584259.1 and WP_099780539.1) was not detected. See Table 2. Results from the STRING database showed that the protein with accession number WP_004584259.1 has neighborhood and co-occurrence interactions with TonB-dependent receptor (HR09_06515) and lipoproteins (HR09_06520). Unfortunately, no annotation or information is available about any of the proteins that interact with WP_099780539.1 (Fig 5).

**Table 2:** Data on conserved domains of putative immunogenic proteins against *P*. *gingivalis* using NCBI Conserved Domain Database, EggNOG and STRING databases.

Accession number	EggNOG	CDD	STRING
**WP_097626800.1**	Inorganic ion transport and metabolism	TonB dependent receptor	Annotation not available
	(Siderophore transport)		
**WP_005874477.1**	Inorganic ion transport and metabolism	TonB dependent/Ligand-Gated channels	Annotation not available
	(Outer membrane protein beta-barrel family)		
**WP_004585254.1**	Inorganic ion transport and metabolism	TonB dependent/Ligand-Gated channels	Annotation not available
	(TonB-dependent receptor)		
**WP_004583657.1**	Inorganic ion transport and metabolism	TonB dependent/Ligand-Gated channels	Annotation not available
	(TonB-dependent receptor)		
**WP_099779133.1**	Inorganic ion transport and metabolism	TonB dependent receptor	Uncharacterized protein
	(Receptor)		
**WP_099840460.1**	Inorganic ion transport and metabolism	TonB dependent receptor	Annotation not available
	(TonB-dependent receptor)		
**WP_211599956.1**	Inorganic ion transport and metabolism	TonB dependent/Ligand-Gated channels	Uncharacterized protein
	(Receptor)		
**WP_012457596.1**	Inorganic ion transport and metabolism	TonB dependent receptor	Annotation not available
	(Receptor)		
**WP_021664214.1**	Lipid transport and metabolism	Long-chain fatty acid transport protein	Uncharacterized protein
	(long-chain fatty acid transporting porin activity)		
**WP_004584259.1**	Function unknown	Not Available	Uncharacterized protein
	(Not Available)		
**WP_099780539.1**	Function unknown	Not Available	Uncharacterized protein
	(Not Available)		
**WP_004583425.1**	Lipid transport and metabolism	Type IX secretion system protein PorQ	Annotation not available
	(long-chain fatty acid transporting porin activity)		

### 3.6. Multi-epitope-based vaccines

**Fig 6:**
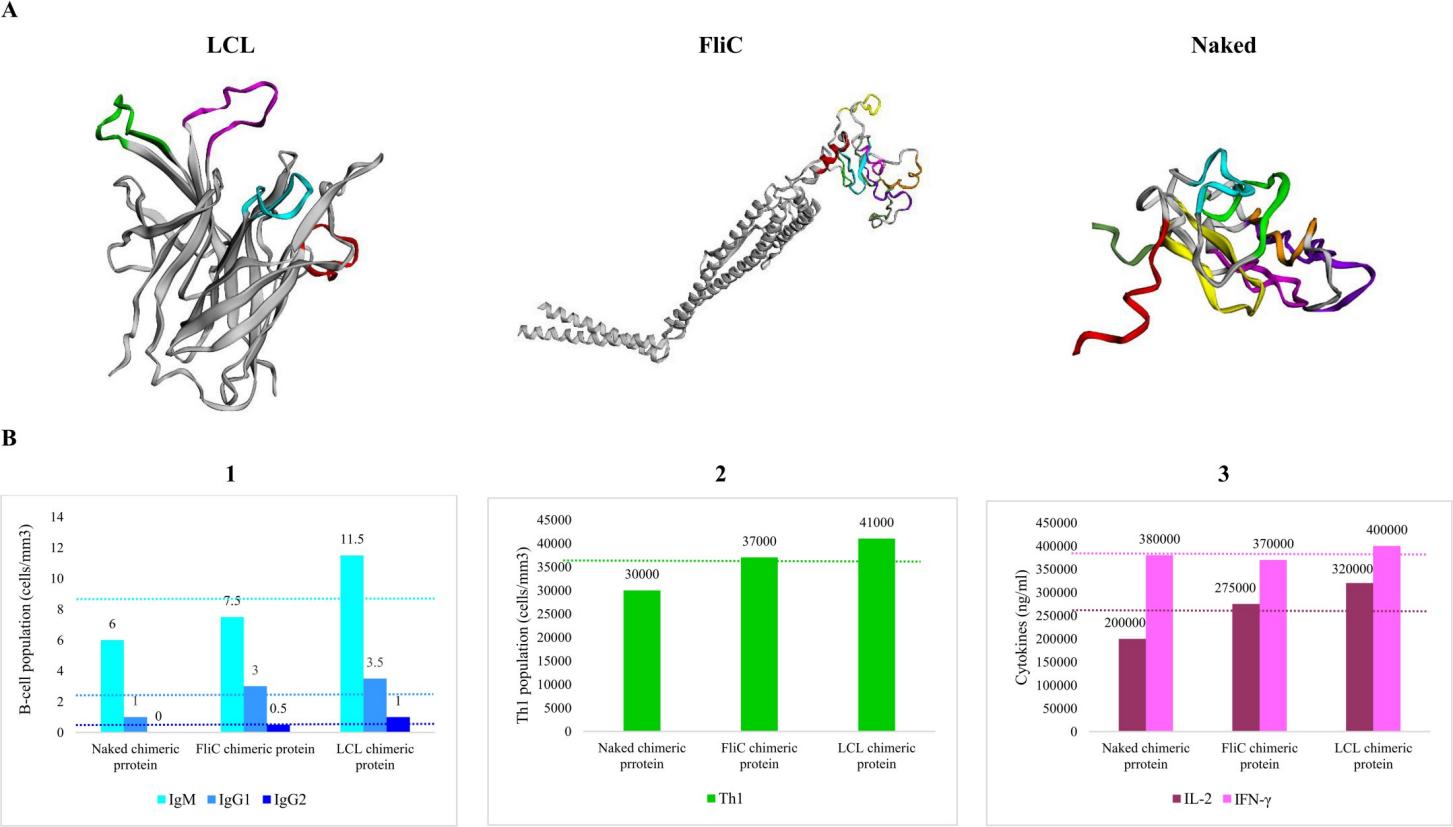
The tertiary structure of multi-epitope vaccines and immune simulation. **A)** 3D structures of the multi-epitope vaccines were predicted by the Robetta webtool and validated by the ProSA-web server. The linear epitopes are colored using Jmol software. **B)** The immunoreactivity of multiple epitope-based proteins was predicted by the C-ImmSim web server. **1.** The levels of the B-cell population secreting IgM, IgG1 and IgG2. **2.** The levels of Th1 populations. **3.** The levels of IL-2 and IFN-γ cytokines. The LCL chimeric protein showed the greatest immunoreactivity.

Eight linear B-cell epitopes with conservation > 80% and antigenicity > 1 were considered to generate multi-epitope vaccines in three platforms, including FliC, LCL, and Naked chimeric protein. The Naked chimera was designed using eight selected epitopes and flexible and rigid linkers. Another multi-epitope vaccine was developed using the same epitopes on the FliC platform. Four epitopes with the greatest conservation and antigenicity were selected to generate an LCL-based chimeric protein. Epitope shuffling was performed, and finally, 24 different arrangements were developed. The antigenicity of these 24 models was evaluated, and the most antigenic arrangement was selected. Finally, three multi-epitope vaccines were achieved. The tertiary structure was validated and shown in Fig 6. The Ramachandran plots denote that more than 90% of the residues of all proteins were located in the favored region. Moreover, the ProSA-web analysis represents that the Z-score of all proteins were in the range of native conformations of the database (S1 Fig).

### 3.7. Dockings with TLRs and immune simulation results

The molecular docking results showed that all multi-epitope-based vaccines had a reasonable binding affinity to the TLR-1, 2, 4, and 6. However, FliC chimeric protein had the strongest affinity to the human TLRs. See Table 3. Based on the results of C-ImmSim, the multi-epitope-based vaccine developed on the LCL platform was shown to provide the most outstanding safety with the strongest stimulation of IgM, IgG1, IgG2, and Th1, cytokines IL -2, and IFN-γ. See Fig 6.

**Table 3:** Data on molecular dockings of multi-epitope vaccines with human TLR 1, 2, 4, and 6.

TLRs	TLR-1				TLR-2				TLR-4				TLR-6			
Protein name	Electrostatics	De-solvation	Van-der Waals forces	Total	Electrostatics	De-solvation	Van-der Waals forces	Total	Electrostatics	De-solvation	Van-der Waals forces	Total	Electrostatics	De-solvation	Van-der Waals forces	Total
**Naked chimeric protein**	-15.735	-2.530	-3.582	-18.623	-29.222	7.316	4.712	-21.436	-6.884	-28.433	46.801	-30.637	-23.824	0.043	-7.181	-24.500
**FliC chimeric protein**	-20.450	-1.102	-24.835	-24.035	-17.910	-15.051	8.155	-32.145	-17.326	-12.505	-10.532	-30.884	-24.601	3.077	1.102	-21.414
**LCL chimeric protein**	-20.695	-6.530	31.954	-24.030	-14.057	-7.566	35.007	-18.122	-9.410	-24.725	13.722	-32.762	-11.572	-14.156	44.530	-21.275

## 4. Discussion

Chronic periodontitis, a multifactorial chronic inflammatory disease due to dysbacteriosis, is characterized by the destruction of connective tissue and alveolar bone and has become the leading cause of tooth loss in adults. It affects almost 50% of the population worldwide and is one of the most common inflammatory diseases in humans [29].

*P*. *gingivalis* is one of the bacteria involved in bacterial plaque biofilm formation and plays a vital role in the progression of chronic periodontitis. In a systematic review, the authors reviewed the literature on *P*. *gingivalis* and all demonstrated systemic implications. From their results, it appears that *P*. *gingivalis* also plays a role in the development of several systemic diseases, including rheumatoid arthritis, cardiovascular disease, and neurodegenerative diseases [30]. This bacterium has also been detected in the brains of patients with Alzheimer’s disease. Dominy and colleagues reported that infection with *P*. *gingivalis* contributes to the pathogenesis of Alzheimer’s disease by secreting gingipains to promote neuronal damage [30]. Therefore, it is important to design and develop a vaccine against this bacterium to fight infections caused by it. To date, several studies have been conducted to develop vaccines against this bacterium. For example, a study by Hyun-Su and colleagues showed that vaccination by *P*. *gingivalis* proteins could prevent atherosclerosis [31]. In addition, in the study by Huang and colleagues, recombinantly produced *P*. *gingivalis* minor fimbriae proteins (Mfa1), RgpA gingipain hemagglutinin domain 1 (HA1), and RgpA gingipain hemagglutinin domain 2 (HA2) were elicited protein-specific IgG [32].

On the other hand, several *in silico* studies have been performed to find effective vaccines against this bacterium. For example, Khan *et al*. considered three antigenic and essential proteins including histidine kinase, Fe (2+) transporter, and capsular polysaccharide transport protein for vaccine design [4]. Finding proteins involved in inorganic ion transport in their and current studies demonstrates the importance of these proteins to potential immunogenicity. Moreover, Santos-Lima *et al*. identified epitopes from Lys-gingipain (Kgp) and neuraminidase virulence factors of the *P*. *gingivalis* ATCC 33277 strain as candidate epitopes [33]. The use of different bioinformatics methods and analyzes can explain this difference in the search for suitable vaccine candidates. However, since no appropriate vaccine is available, studies are currently underway to find a new vaccine candidate.

With the advent of genome sequencing technology, the need to culture bacteria has diminished and been replaced by reverse vaccinology techniques. There are several predictive and analytical software programs such as Vaxign and VaxiJen that use the reverse vaccinology approach. These software programs were developed to identify potential vaccine candidates [34]. This present study is significant in that we considered 17 genomes of different *P*. *gingivalis* strains and used a core proteome approach to develop a suitable vaccine candidate. The core proteome presents the most similar and common proteins between different bacterial strains. In addition, we used the quartile method of scoring in this study to find suitable targets. It should be mentioned that quartile analysis is a valuable approach in bioinformatics studies. This is because different criteria such as antigenicity, allergenicity, adhesion probability, *etc*. can be considered simultaneously in selecting the best vaccine candidates.

This comprehensive study has shown that of the 1418 proteins of *P*. *gingivalis*, only 39 proteins are exposed at the surface. Therefore, we analyzed them from different aspects to select the best putative immunogenic targets. For example, only proteins with a molecular weight of < 110 kDa were selected because proteins with such molecular weight are desirable for protein purification and *in vitro* evaluations [35]. Moreover, the physicochemical properties of putative immunogenic targets are essential factors for the optimal selection of a protein. This is because these properties directly determine the biological behavior of the peptide and influence other vaccine-related processes. Adhesion proteins are considered critical vaccine candidates because they elicit host cell responses and mediate bacterial invasion. Therefore, adhesion probability is considered an appropriate criterion for prioritizing candidates in the reverse vaccinology approach [36]. In this study, we also consider only virulence factors as suitable candidates because virulence is one of the most important properties of the vaccine. Virulent proteins are more likely to initiate infection pathways compared to non-virulent proteins [37].

This study presented 12 immunogenic targets and three multiple epitope-based vaccines against *P*. *gingivalis* that are non-homologous to human proteins. The results of the protein domain search showed that most of the selected proteins were involved in the transport and metabolism of inorganic ions and the transport and metabolism of lipids; the function of two proteins (WP_004584259.1 and WP_099780539.1) was not detected. However, the STRING database showed that the protein with accession number WP_004584259.1 has interactions with TonB-dependent receptors and lipoproteins. Unfortunately, no interaction was detected for WP_099780539.1, and the role of this protein remained unknown.

It was approved that several scarce nutrients, such as iron and nickel, are essential for bacterial growth. Gram-negative bacteria secrete chelators to competitively bind these nutrients from the environment. Transport of the resulting complexes into bacterial cells is mediated by TonB-dependent transporters (TBDTs), which are located on the outer membrane in Gram-negative bacteria. The properties of TBDTs, such as surface exposure, protective immunogenicity, wide distribution, inducible expression *in vivo*, and essential role in pathogenicity, make them excellent candidates for vaccine development [38, 39]. However, we should consider that TBDTs are subject to high selection pressure due to their surface position and key role in virulence, resulting in frequent variations in some TBDTs. Therefore, a single TBDT antigen is sometimes insufficient for vaccine development [40].

To solve this problem, the development of epitope-based chimeric/subunit vaccines may be helpful. Epitope-based chimeric/subunit vaccines have many advantages over vaccines produced by conventional vaccinology. For example, they are inexpensive to develop, do not require microbial culturing, and can outperform many wet-lab experiments with saving time. They are a safer option because they do not contain the entire pathogen and are highly specific and stable [41]. Of the three multiple epitope-based vaccines presented in this study, the vaccine developed on the LCL platform emerged as the one with the greatest safety and strongest stimulation of IgM, IgG1, IgG2, Th1, cytokines IL-2, and IFN-γ based on the results of C-ImmSim, making it perhaps the most desirable vaccine candidate.

The subtractive proteomics and reverse vaccinology approaches performed by Khan *et al*. presented three vaccine candidates, including histidine kinase, Fe transporter, and capsular polysaccharide transport protein. Fe transporter was the common protein identified in the present study and its investigation. However, we did not identify histidine kinase and capsular polysaccharide transport protein. The use of different bioinformatics approaches and pipelines may justify this difference [4].

## 5. Conclusion

This study investigated the novel immunogenic targets against *P*. *gingivalis* using reverse vaccinology, immunoinformatic analyses, and computer-aided approaches. Twelve novel vaccine candidates are proposed. Moreover, three multi-epitope vaccines were generated using Naked, LCL, and FliC platforms. Among three multi-epitope vaccines, FliC chimeric protein had the strongest affinity to the human TLRs while the LCL platform induced the highest level of immunoglobulins, cytokines, and Th1 response. The results of this study could help find an effective vaccine against this pathogen. This study might establish the fundamental of vaccine development against this pathogen to prevent periodontitis. However, experimental validation through *in vitro* and *in vivo* assays are necessary to confirm the safety and immunization of proposed vaccine candidates and multi-epitope vaccines.

## Supporting information

S1 Table Physicochemical properties of 26 putative immunogenic proteins. (XLSX)

S2 Table The conservancy of linear and conformational B cell epitopes among *P. gingivalis* strains. (DOCX)

S1 Fig Ramachandran plots and ProSA-web analysis of three multi-epitope constructs. (TIF)
