# Supplementary material for: Reverse vaccinology approach to identify novel and immunogenic targets against Porphyromonas gingivalis: An in silico study
Source: PLoS One. 2022 Aug 30;17(8):e0273770. doi: 10.1371/journal.pone.0273770 (PMC9426909; doi:10.1371/journal.pone.0273770)
Supplement: S2 Table — (DOCX) [file pone.0273770.s002.docx]

**Supplementary data 2.** The conservancy of linear and conformational B cell epitopes among *P. gingivalis* strains

| **Protein name** | **start- End** | **Linear B cell epitopes** | **Conservancy %** | **Conformational B cell epitopes** | **Score** | **Conservancy %** | **Colo r in 3D Structure** |
| --- | --- | --- | --- | --- | --- | --- | --- |
| WP_097626800.1 | 164 -170 | SNSLTGF | 100 | M1, K2, K3, N4, G5, L6, S7, Y8, I9, P10, V11, L12, R13, A14, L15, L16, I17, C18, T19, L20, L21, L22, L23, V24, Y25, G26, A27, S28, A29, Q30, H31, H32 | 0.97 | 50 | - |
|  | 179 - 185 | GSDDNTS | 100 |  |  |  |  |
|  | 217 - 232 | LYVADRTVTRRTGMNA | 100 | G422, P423, E434, H435, E436, G437, V438, F439, S442, F610, V612, L613, N614, A615, Y616, M617, Q618, N619, P620, E621, L622, D623, H624, L625, I626, D685, Y687, T688, K689, L690, I691, A692, P693, L694, S695, P696, G697, H698, K699, P700, Q701, E703, D704, Y705, I706, Q707, H709 | 0.899 | 20.83 | Red |
|  | 316 - 339 | PDVREVLDSYKRSTLHVRYSDQLN | 25 |  |  |  |  |
|  | 418 - 436 | ISTSGPKSMPVATESGEHE | 100 | I467, N468, F469, T470, E471, R472, H473, H474, H475, S532, I533, E534, W535, G536, A537, N538, R539, L540, W574, Y576, R577, A578, E579, H580, L581, I583, Y655, K656, R657 | 0.88 | 87.5 | - |
|  | 501 - 509 | PYPGNNSYI | 95.83 |  |  |  |  |
|  | 554 - 560 | LPSSYVL | 50 | V33, S34, T35, C36, T37, I38, R39, G40, K41, V42, T43, M45, A46, G47, K48, E49, G50, I51, G52, F53, A54, T55, V56, L58, A59, D60, Q61, A62, Y63, G64, V65, A66, C67, D68, G69, K70, G71, E72, F73, V74, I75, K76, R77, V78, T79, A80, G81, S82, Y83, C89, L99, H100, I101, K102, A103, D104, T105, T106, V107, H108 | 0.873 | 83.33 | - |
|  | 681 - 712 | PIAFDRYTKLIAPLSPGHKPQKEDYIQEHHKD | 20.83 |  |  |  |  |
|  | 764 - 774 | MFRPIVSEYEQ | 100 | N339, G341, D342, S391, G392, S393 | 0.839 | 100 | - |
|  | 912 - 921 | YKQANNTTER | 100 | S847, K848, A849, L852, E853, A856, T857, N858 | 0.808 | 100 | Blue |
| WP_005874477.1 | 252 - 258 | QRYMAGG | 91.67 | G910, N911, M912, N913, R914, P915, G916, P917, P918, F919 | 0.972 | 100 | - |
|  | 277 - 313 | NTNNMGFSEMDSEMGSMTFFSPQGGGRRGFGNSGGVT | 54.17 | R2, S3, I4, Y5, Q6, L7, L8, L9, S10, I11, L12, L13, A14, S15, L16, G17, F18, V19, G20, L21, E22, A23, Q24 | 0.967 | 41.67 | - |
|  | 402 - 417 | IDGFFNDTYETKDATG | 100 | Q25, A26, G27, V28, A29, G30, R31, V32, L33, D34, E35, E36, G37, N38, P39, M40, I41, Q42, A43, N44, V45, L47, V48, Q49, S50, T51, G52, Q53, V54, A57, A58, G59, A60, T61, N62, E63, K64, G65, L66, F67, S68, L69, K70, T71, S72, Q73, E74, G75, D76, Y77, I78, L79, R80, V81, S82, Y83, V84, G85, Y86, T87, T88, H89, D90, E91, K92, I93, S94, L95, R96, N97, G98, Q99, T100, I101, T102, L103, K104, D105, I106, S107, M108, N109, E110, D111, A112, R113, L114, L115, Q116, S117 | 0.897 | 95.83 | - |
|  |  |  |  | K413, D414, A415, T416, G417, I418, S419, I420 | 0.873 | 83.33 | Red |
|  | 471 - 479 | QAVLQSVET | 83.33 | A897, G902, R904, S905, D906, H907, Q908, R909, G920, G921, G922, R923, R924, P925, S926 | 0.865 | 100 | - |
|  | 530 - 549 | DDGQYSILDSQYGLSYSNEF | 87.5 | L528, G529, D530, D531, G532, Q533, S535, I536 | 0.852 | 87.5 | Blue |
|  | 692 - 697 | YDPSTG | 100 | L355, A356, E357, G358, N359 | 0.849 | 100 | Green |
|  | 787 - 792 | SGQKDS | 95.83 | Q475, S476, V477 | 0.826 | 100 | Yellow |
|  | 822 - 828 | NSGYSGG | 95.83 | S539, V584, A585, G586, V587, E588, Q589 | 0.812 | 100 | Pink |
|  | 898 - 917 | FSGGGSRSDHQRGNMNRPGP | 100 | K384, P385, S386, E387, R388, H444, L446, N447, D448, E449, G450, R451, E500, P501, L502, G503 | 0.812 | 79.17 | - |
| WP_004585254.1 | 291 - 305 | YRNHWDANNDGYSEL | 100 | M1, I2, G3, K4, K5, I6, F7, F8, I9, L10, L11, A12, L13, I14, A15, F16, S17, G18, L19, N20, A21, A22, T23, D24, T25, E26 | 0.976 | 62.5 | - |
|  | 339 - 362 | RGGDRIDLPPHVVGVAEQTDHSVF | 100 | F27, K28, Y29, P30, T31, D32, A33, N34, I35, I36, G37, H38, H47, I55, K56, G57, T58, T59, F60, D65, A66, T67, G68, H69, Y70, Y71, L72, R73, N74, L75, R76, P77, G78, E79, I80, T81, L82, R94, V95, V96, R97, V98, E99, K100, D101, K102, T103, I104, E105, V106, N107 | 0.901 | 100 | - |
|  | 406 - 426 | PGGTEGYPIPQDQYGNNYGVT | 100 | F444, L445, L446, M447 | 0.867 | 100 | - |
|  | 468 - 487 | ILSWQTGEDANGNTIPLYPE | 100 | I400, D401, V402, N403, G404, H405, P406, G407, G408, T409, E410, G411, Y412, P413, I414, P415, Q416, Q418, S470, Q472, T473, G474, E475, D476, A477, N478, G479, N480, T481, I482 | 0.865 | 100 | Red |
|  | 623 - 627 | PDQHD | 100 | L677, N678, T679, V680, K681, D682, T683, N684, G685, A686, F687, V688, T689, E690, A691, N692, A693, N694, G695, Q696, Q697, E698, Y699, K700, N701, E702, M759, Q760, N761, N762, P763, E764, I765, T766, D767, E768, T769, G770, T771, A772, P773 | 0.853 | 70.83 | Blue |
|  | 671 - 709 | EAQEWGLNTVKDTNGAFVTEANANGQQEYKNESMTDTQI | 100 |  |  |  |  |
|  | 747 - 776 | IEYGVKSAELDIMQNNPEITDETGTAPRID | 70.83 |  |  |  |  |
|  | 819 - 829 | DFDRGAARDSG | 100 | H796, V797, F798, A800, T801, F848 | 0.845 | 100 | - |
| WP_004583657.1 | 257 - 261 | KGWGS | 100 | L16, G17, T18, S19, A20, S21, A22, Q23, Q24, S25, G26, G27, S28, V29, T30, G31, V49, K50, G51, T52, T53, L54, S65, I66, K67, G68, I69, P70, S71, G72, N73, Q74, T75, I76, E87, E88, K89, V90, H91, I92, E93, K94, G95, G96, S97, R98, H99, V100, D101, Y103 | 0.924 | 95.83 | - |
|  | 289 - 302 | YRPGQDIDGDSFTE | 70.83 |  |  |  |  |
|  | 341 - 350 | RLDNPPFEAQ | 100 | E616, T617, T618, S619, D620, G621, K622, E623 | 0.888 | 100 | Red |
|  | 395 - 419 | GDYTENLLNGAVQSGSTESDEYNDA | 41.67 | E348, Q350, E399, N400, L401, L402, N403, G404, A405, V406, Q407, S408, G409, S410, T411, E412, S413, D414, E415, Y416, N417, D418, A419, F420, T424, D677, K678, T679, T680, G681, Q682, A683, G731, D732, I733, P734, A735, E736, H737, I738, A739, S740, D741, G742, S743, F744, D745, F746, E747, M748, N749, G750, Q751, Q752, F753, K754, G755, L756, A757, E758, H760 | 0.871 | 8.33 | Blue |
|  | 462 - 472 | DDRSGYRPSKI | 100 |  |  |  |  |
|  | 550 - 559 | DEDLHVELAG | 100 | D508, G509, K510 | 0.849 | 100 | Yellow |
|  | 614 - 623 | KVETTSDGKE | 100 | T489, E490, K491, L492, Y527, N528, P529, N530, K531, N532, L533 | 0.843 | 100 | - |
|  | 672 - 691 | TAVEADKTTGQAEISVKDYV | 95.83 | K374, D375, H442, T443, F444, G445, E446, N447, W448, L485, Y487 | 0.841 | 100 | - |
|  | 725 - 766 | VHEAYEGDIPAEHIASDGSFDFEMNGQQFKGLAEGHAKLVKT | 8.33 | Y502, Y512, I513, D514, P515, F517 | 0.82 | 100 | Green |
|  | 803 - 813 | KDTDKGPGRAS | 95.83 | H255, A256, K257, G258, W259, G260, Q293, D294, D296, G297, D298 | 0.809 | 100 | Orange |
| WP_099779133.1 | 146 - 152 | RVNAGPT | 100 | K8, N9, K10 | 0.969 | 100 | - |
|  | 199 - 207 | LVRSAQQEG | 100 | M1, A4, I5, P6, R7, Y11, I12, K13, L14, N15, G16, I17, Y18, R19, L20, S21, F22, I23, L24, L25, C26, C27, L28, L29, C30, S31, Q32, A33, A34, M35, A36, Q37, S38, V39, R40, V41, K59, V60, R61, G62, T63, G64, T65, R78, M79, K80, A81, T82, T83, D84, S85, I86, T87, I88, S101, F102, P103, S104, L105, T106, K107, D108, T109 | 0.926 | 12.5 | - |
|  | 261 - 268 | LVRSAQQEG | 100 | V711, P712, G713, N714, K715, R716, I717, Y757, K758, W759, L760, D761, P762, D763, D764, S765, F766, A767, G768, R769, S770, K771, W772, L773, M774, G775, V776, K777, G778, A779, F827 | 0.9 | 62.5 | - |
|  | 289 - 295 | DTKAEYD | 100 | G338, T339, L340, S341, D342, A343, D402, V403, Q404, L405, G406, A407, D408, G409, T410, A411, S412, M413, A414, S415, G416, S417, E418, N419, S420, N421, G422, L423 | 0.864 | 70.83 | Red |
|  | 389 - 427 | RETYDIQGEYFLNDVQLGADGTASMASGSENSNGLGIGR | 83.33 | N496, K584, D585, A586, E587, G588, N589 | 0.863 | 100 | Blue |
|  | 477 - 491 | RRDSVGYNLPHSETV | 95.83 | D479, S480, V481, G482, Y483, N484, L485, P486, H487, S488, E489, T490, V491, L492, L493, M494 | 0.847 | 95.83 | Yellow |
|  | 582 - 588 | THKDAEG | 87.5 | E121, L122, S123, S124, V125 | 0.821 | 100 | - |
|  | 733 - 742 | NPSKGLSSPA | 95.83 | Q96, G97, A116, A118, E119, M120 | 0.803 | 100 | - |
| WP_099840460.1 | 20 - 28 | NSEIDSLSN | 100 | D606, A607, D608, Y609, N610 | 0.934 | 100 | Red |
|  | 253 - 269 | DEAKYGRRYNSAGLMYL | 8.33 | M1, I2, A3, A4, L5, A6, V7, L8, P9, F10, C11, L12, T13, A14, Q15, A16, L17, V18, F19, N20, S21, E22, I23, D24, S25, L26, S27, N28, V29, L31, L162, A163, P164, S186, G187, R188, I189, G190, R191, H192, W193, G226, S227, N228 | 0.914 | 8.33 | - |
|  | 273 - 282 | GVPHYYHNTD | 91.67 | L325, K326, A329, L330, Q331, P332, Y333, V334, E335, N336, S337, V338, T339, V340, K341, Y390, I391, K392, Y394, N395, Q396, P397, L398, A399, P400, D401, F402 | 0.891 | 100 | Blue |
|  | 323 - 341 | RKLKEYALQPYVENSVTVK | 100 | W575, I577, L578, P579, R580, L581, L582, L638, S639, H640, A641, G642, F643, M645, G678, Y679, V680, L681, P682, V683, H684, F685, V686, K687, R688, V689, L691, S740, L741, T742, I743, D744, F745 | 0.87 | 83.33 | - |
|  | 391 - 406 | IKKYNQPLAPDFEYYR | 87.5 |  |  |  |  |
|  | 447 - 462 | TDEYDEVQGSMQHIDL | 100 | I424, T425, P426, E427 | 0.855 | 100 | - |
|  | 497 - 505 | NRTNYTEAG | 100 | G265, Y268, L269, D270, A271, Q272, G273, V274, P275 | 0.844 | 8.33 | Green |
|  | 600 - 621 | QYTSVYDADYNWLELKEETLES | 95.83 | R597, V599, Q600, Y601, V604, Y605, W611, L612, E613, L614, K615, E616, E617, T618, L619, E620, S621, V715, Q716, A717, S718, G719, E720, L721, S722, A723 | 0.829 | 87.5 | Yellow |
|  | 658 - 665 | DNTQRSDR | 95.83 |  |  |  |  |
|  | 714 - 722 | YVQASGELS | 91.67 | Y450, D451, E452, V453, Q454, G455, S456, M457, Q458 | 0.822 | 100 | Orange |
| WP_211599956.1 | 277 - 305 | LLLKPEYKLPGTNRSYAYNARTIWNPTSQ | 41.67 | M1, L4, I5, S6, R7, T8, L9, I10, W11, L12, L13, L14, I15, L16, C17, I18, P19, I20, S21, L22, A23, A24, Q25, R26, Q27, I28, A29, G30, T31, T33, D34, T35, K36, G37, R38, P39, I40, P47, D57, S58, L59, G60, H61, R63, T65, T66, S67, S68, T69, F70, P71, I72, L74, D84, T85, F86, T87, I88, R89, K90, G91, D92, P93, M94, E95, N96, I97, R98, I99, R100, L101, R102 | 0.911 | 0 | - |
|  | 376 - 387 | LEIEPDIESTFR | 100 | C617, A618, P619, I620, S621 | 0.891 | 100 | - |
|  | 416 - 431 | YSEAKSSVREDKSSAL | 91.67 | F665, K666, Y667, K668, K669, A670, G671, G723, I724 | 0.874 | 100 | - |
|  | 591 - 603 | QYLFSPDQERPDF | 100 | R324, I325, P326, S327, P328, A329, T330, A331, Y332, L376, E377, I378, E379, P380, D381, I382, E383, D638, N640, I641, A642, S643, P644, A645, Y646, L647, N648, G693, K695, P696, I697, G698, G699, V700, Y701, P702 | 0.85 | 70.83 | Red |
|  | 639 - 644 | PNIASP | 70.83 | R424, D426, K427, S428, S429, A430, L431, P432, T433, R435, G506 | 0.846 | 75 | Blue |
|  | 726 - 736 | RRQEIMNTDIK | 100 | S237, H254, L255, S256, R257, S258, N259, R260, P302 | 0.83 | 91.67 | - |
|  |  |  |  | E572, L573, I574, R575 | 0.815 | 8.33 | - |
| WP_012457596.1 | 29 - 37 | AQPTDTIVS | 100 | L302, I303, K304, T305, E306, N307, Q308, K309, L311 | 0.916 | 100 | Red |
|  | 218 - 227 | QFEQELNVAG | 100 | L510, P511, Y512, G513, F514, Y553, G554, H555, N556, F557, S558, K559, N560, Y561, R562, L563, N603, F605, Y606, R607, N608, Y609, V642, T645 | 0.902 | 100 | - |
|  | 257 - 268 | KQHWTDKIDFLY | 100 | G197, I198, F199, I239, S240, P241, T242, E243, N244 | 0.889 | 100 | - |
|  | 306 - 311 | ENQKKL | 100 | K2, S3, V4, V5, T6, K7, Q8, A9, L10, I11, G12, L13, L14, F15, F16, S17, I18, S19, I20, Y21, S22, H23, A24, A25, N26, P27, P28, A29, Q30, P31, I281, S282, E283, T284, L329, A330, E331, V332 | 0.886 | 100 | - |
|  | 349 - 359 | PRLSSPNDPGE | 100 | A529, T530, D531, K532, N581, S582, N583, E584, L585 | 0.886 | 75 | Blue |
|  | 431 - 435 | FFNHG | 100 | S352, S353, P354, N355, D356, P357, G358, F431 | 0.873 | 100 | Orange |
|  | 530 - 536 | TDKAGTE | 100 | N433, F438, K484, G485, T486, D487 | 0.873 | 100 | Yellow |
|  | 576 - 587 | KTGRMNSNELFE | 100 | D170, P171, F172, V194, R196 | 0.864 | 100 | - |
|  |  |  | 100 | V375, T376, P377, K378, C407, S408, H409, W461 | 0.855 | 95.83 | - |
|  |  |  | 75 | D217, Q218, F219, K624 | 0.848 | 100 | Cyan |
| WP_021664214.1 | 101 - 110 | QGSSETFNKN | 100 | A382, G383, K384, I385, L432, R433, P434, I435, S436, R437, L438, L440, L489, Y491, R492, F493, T494, N496, L497, Y552 | 0.916 | 100 | - |
|  | 169 - 181 | GKANPSHFNYNGL | 100 | Y211, K212, Q213, N214, G215, E216, Y217, Y336, S338, Q339, G340, V341, D342, S343, N344, G345, Q346, P347, L348, P349, E350, N351, L352, D521, D522, P523, N524, P525, A526, N527, V528 | 0.901 | 100 | Red |
|  | 212 - 222 | KQNGEYQIFGP | 100 | G124, K125, A126, N127, E128, D129, V130, I246, Q247, D248, T249, W250, Y305, P307, A308, D309, A310 | 0.9 | 100 | - |
|  | 270 - 281 | QENFSFNNGAIN | 100 | S274, F275, N276, N277, G278, A279, I280, N281, D282 | 0.897 | 100 | Blue |
|  | 338 - 362 | SQGVDSNGQPLPENLYFMSSQTPES | 100 | P168, K170, A171, N172, S174, H175 | 0.849 | 100 | Yellow |
|  | 404 - 417 | DENGTAYVDNKFIS | 100 | L199, P200, G201, G202, Y203, G204 | 0.839 | 100 | Cyan |
|  | 457 - 466 | KAFDGSAQVT | 62.5 | K403, D404, E405, N406, G407, T408, A409, Y410 | 0.809 | 100 | Orange |
|  | 475 - 479 | HYELP | 100 | N177, Y178, N179, G180, L181, E182 | 0.809 | 100 | Green |
|  | 517 - 533 | RMVSDDPNPANVLEVES | 100 |  |  |  |  |
| WP_004584259.1 | 84 - 96 | YGDIAGDYLPYNG | 58.33 | R382, T383, D384, R385, G386, C387, H426, A427, G428, S429, K430, L431 | 0.913 | 45.83 | - |
|  | 190 - 197 | LTDPRTTN | 100 | D74, S75, I110, S111, V112, R113, N114, Y115, G116, Y167, F169, R170, A171, G172, R173, L174, P175, L176, L210, T211, L212, P213, R214, E215, S216, R282, R283 | 0.904 | 8.33 | - |
|  | 229 - 239 | QHLTQYNWRPG | 100 | R301, S302, S303, I304, I342, E344, D345, Y346, K347, P348, D349, D350, N351, Y352, I354, Y355, D356, L357, R358, I359, L360, A361, I362 | 0.897 | 100 | Red |
|  | 528 - 532 | QAERT | 100 | M1, K2, T3, I4, R6, P487, M488, Q489, N490, T491 | 0.895 | 75 | - |
|  |  |  |  | L322, S323, G324, Y379 | 0.857 | 62.5 | - |
|  |  |  |  | H407, G408, H409, H410, Y449, K450, I451, R452, T453, I454, Q455, K456 | 0.843 | 100 | Blue |
|  |  |  |  | T191, D192, P193, N235, R237, P238, G239, Q240, Q241, D242, S256, N257, S258, P259, I260, W261, H353 | 0.839 | 100 | Yellow |
|  |  |  |  | G514, K515, T516, P517 | 0.81 | 100 | Cyan |
| WP_099780539.1 | 47 - 60 | QVKDRQNQEDGYGD | 16.67 | S383, S384, S385, S386, G387, S388, M389, S390, G391, G392, G393, G394, R395, S396, G397, R398, G399, R400, N401 | 0.975 | 58.33 | Red |
|  | 73 - 85 | DAYNRRDGQSYDG | 100 | S366, T367, P368, S369, R370, S371, N372, S373, N374, G375, G376, F377, S378, T379, P380, S381, R382 | 0.906 | 33.33 | Blue |
|  | 88 -102 | LSKDKKRDSTRSSVP | 100 | R319, N320, I321, E322, T323, V324, T325, P326, N327, N328, G329, Q330, K331, Q332 | 0.89 | 91.67 | Green |
|  | 266 - 276 | IKGGTSDAKLG | 16.67 | T312, G313, R314, A315, N316, R317 | 0.889 | 100 | Yellow |
|  | 283 - 318 | IQKSSSQKNKFGLQSNKPNNNLQNVKPGRTGRANRD | 4.17 | I72, D73, A74, Y75, N76, R77, R78, D79, G80, Q81, S82 | 0.843 | 100 | Cyan |
|  | 327 - 363 | NNGQKQNRPVFQQNQSGNNRPTGRNIRSERQGENNDR | 37.5 | P309, G310, R311 | 0.838 | 100 | Purple |
|  | 366 - 397 | STPSRSNSNGGFSTPSRSSSSGSMSGGGGRSG | 8.33 | P300, N302, L304, Q305, N306, V307, K308 | 0.812 | 95.83 | Pink |
| WP_004583425.1 | 130 - 139 | TFDELGESMG | 100 | V199, L200, L201, G202, N203, A204, E205 | 0.943 | 100 | Red |
|  | 174 - 184 | STHNTGENQAG | 100 | M1, I2, I3, K4, K5, M6, L7, K8, N9, K10, L11, A12, A15, L19, F20 | 0.921 | 91.67 | - |
|  | 290 - 302 | ISGIFSSFGDAPG | 95.83 | I268, M269, D270, Q271, N272, D273, Q274, A275, G276, E278, A279, A280, K282, K283 | 0.901 | 100 | - |
|  |  |  |  | L108, G109, T110, D111, E112, N113 | 0.873 | 100 | Blue |
|  |  |  |  | Y244, I246, D247, D248, Y249, N250, D322, D323, K324 | 0.867 | 100 | Yellow |
|  |  |  |  | E129, T130, F131, D132, E133, L134, G135, E136, S137, M138, G139, T175, H176, N177 | 0.829 | 100 | - |
|  |  |  |  | G179, E180, N181, G225 | 0.828 | 100 | - |
|  |  |  |  | G298, D299, A300, P301, G302, G303, L304, K305 | 0.815 | 100 | - |
|  |  |  |  | T78, K79, M352, N353, I354, D384, G385, L386, R387, N388, H391 | 0.802 | 100 | Cyan |
