# Supplementary figures and images for: Reverse vaccinology approach to identify novel and immunogenic targets against Porphyromonas gingivalis: An in silico study
Source: PLoS One. 2022 Aug 30;17(8):e0273770. doi: 10.1371/journal.pone.0273770 (PMC9426909; doi:10.1371/journal.pone.0273770)

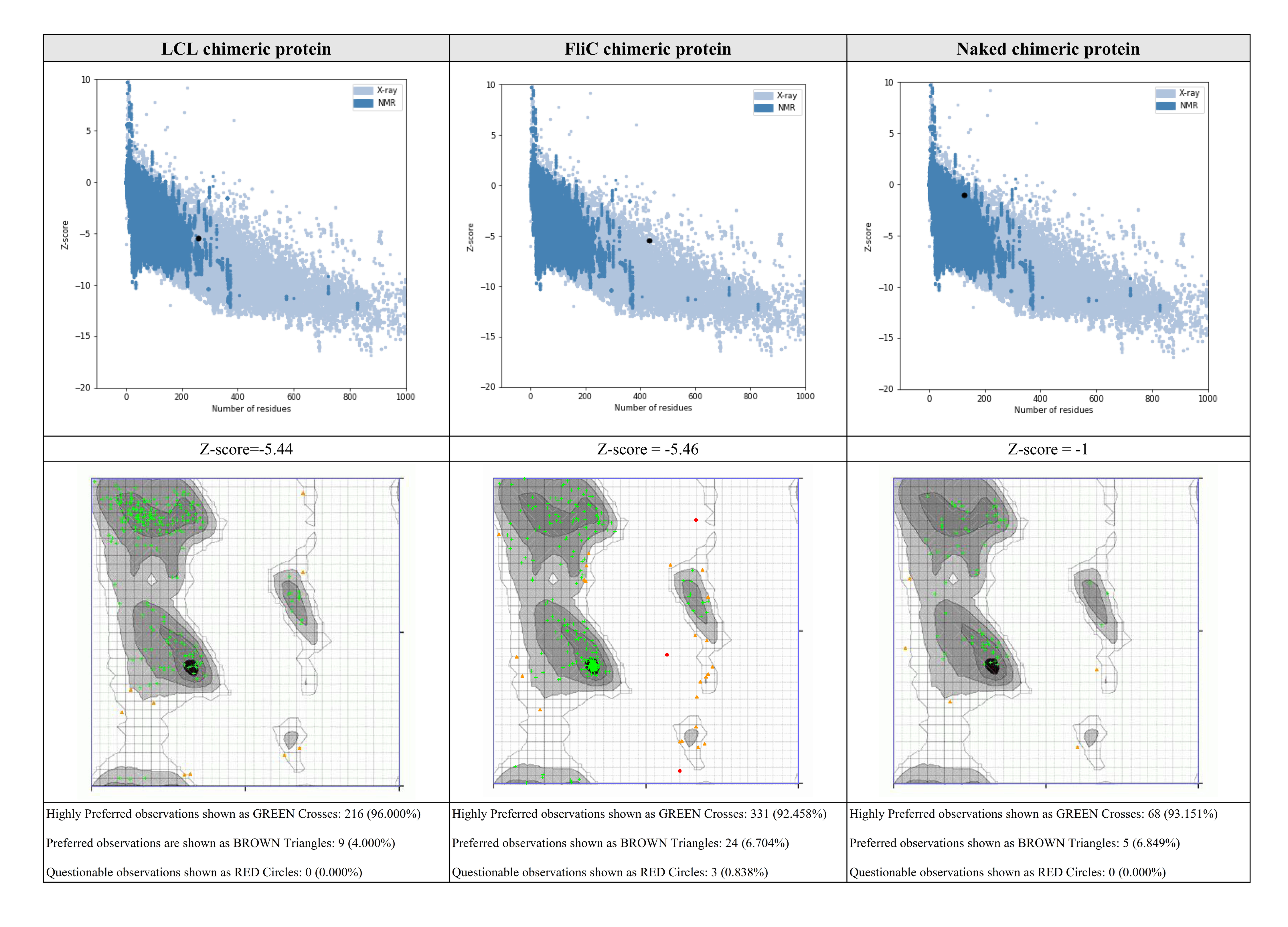

Supplement: S1 Fig — (TIF) [file pone.0273770.s003.tif]
